# Validation of the 30-Year Framingham Risk Score in a German Population-Based Cohort

**DOI:** 10.3390/diagnostics12040965

**Published:** 2022-04-12

**Authors:** Susanne Rospleszcz, Fabian Starnecker, Birgit Linkohr, Moritz von Scheidt, Christian Gieger, Heribert Schunkert, Annette Peters

**Affiliations:** 1 Department of Epidemiology, Institute for Medical Information Processing, Biometry and Epidemiology, Medical Faculty, Ludwig-Maximilians-Universität München, 80539 Munich, Germany; peters@helmholtz-muenchen.de; 2 Institute of Epidemiology, Helmholtz Zentrum München, German Research Center for Environmental Health, 85764 Neuherberg, Germany; birgit.linkohr@helmholtz-muenchen.de (B.L.); christian.gieger@helmholtz-muenchen.de (C.G.); 3 German Center for Cardiovascular Disease (DZHK), Partner Site Munich Heart Alliance, 80636 Munich, Germany; fabian.starnecker@tum.de (F.S.); moritz.scheidt@tum.de (M.v.S.); schunkert@dhm.mhn.de (H.S.); 4 Department of Cardiology, Deutsches Herzzentrum München, Technische Universität München, 80333 Munich, Germany; 5 Research Unit of Molecular Epidemiology, Helmholtz Zentrum München, German Research Center for Environmental Health, 85764 Neuherberg, Germany

**Keywords:** risk prediction, risk factors, cardiovascular disease, cohort study, calibration

## Abstract

The Framingham Risk Score to predict 30-year risk (FRS30y) of cardiovascular disease (CVD) constitutes an important tool for long-term risk prediction. However, due to its complex statistical properties and the paucity of large population-based cohorts with appropriate data, validation of the FRS30y is lacking. A population-based cohort from Southern Germany (N = 3110, 1516 (48.7%) women) was followed up for a median time of 29.5 [18.7, 31.2] years. Discrimination and calibration were assessed for the original, recalibrated and refitted FRS30y version. During follow up, 620 incident CVD events (214 in women) occurred. The FRS30y showed adequate discrimination (original and recalibrated version: Area under the curve (AUC): 78.4 for women and 74.9 for men) but overestimated actual CVD risk (original version: discordance 45.4% for women and 37.3% for men, recalibrated version: 37.6% and 28.6%, respectively). Refitting showed substantial improvement in neither discrimination nor calibration. The performance of FRS30y is adequate for long-term CVD risk prediction and could serve as an important tool in risk communication, especially for younger audiences.

## 1. Introduction

Cardiovascular disease (CVD) is a major source of mortality and morbidity in industrialized and developing countries [1]. Accurate CVD risk assessment is necessary not only for individual counseling, but also for improved guidance of preventive measures. Thus, predictive scores evaluating which individuals are more likely to develop CVD constitute important information for personalized health care decisions, as well as public health policies and guidelines.

CVD risk scores are established tools to achieve this kind of risk assessment. Although a plethora of CVD risk scores exist in the scientific literature [2], only a few have been thoroughly validated and are thus recommended by official medical societies. Among those are, for instance, the Framingham Risk Score (FRS), the Pooled Cohort Equations (PCE) or the Systematic Coronary Risk Evaluation (SCORE) [3,4,5].

External validation of all risk scores in populations different from their derivation cohort is crucial to determine generalizability and applicability. Moreover, subsequent assessments are vital in detecting potential trends and evaluating subgroup-specific performance. Our group already identified susceptibilities of the FRS and PCE to temporal changes in risk factor profiles and found sex-specific trends in the risk scores’ discrimination and calibration over time [6].

Currently, recommendations for CVD prevention are shifting from primary prevention to primordial prevention [7]. As a tool for long-term CVD risk prediction, a risk score was developed from data of the Framingham Heart Study (“FRS30y”), aiming at a younger target group with the goal to forecast their probability of developing CVD over a 30-year horizon [8]. Communicating disease risk at an earlier age is expected to lead to more risk awareness, individual changes towards healthier and more favorable behavior and better adherence to therapy and medication. The FRS30y might therefore serve as an important tool for risk communication for younger age groups.

Due to the long-term horizon of prediction, the methodological setup of the sta-tistical model of FRS30y is more complex compared to traditional scores, which typically only make predictions within a 10-year life span. During a 30-year follow-up, many participants will drop out due to non-CVD death. The commonly used Cox or Weibull model cannot account for that, as these people would still be treated as if they are under risk for CVD. For accurate risk prediction, a model accounting for these competing non-CVD deaths is therefore necessary.

The FRS30y is based on such a competing risk model, incorporating survival and censoring information for both CVD and competing events, iterating over event times. Both an online calculator and an Excel spreadsheet are available to calculate a single individual’s 30-year CVD risk. However, due to the iterative structure of the calculation, there is no closed formula of the FRS30y. This limitation and the fact that only few population-based cohorts have high-quality data with long-term follow-up makes an adequate recalibration assessment of the FRS30y challenging. However, such recalibration will give important insights into generalizability and applicability of the score.

There are different methods to update an existing prediction model for a sample of new individuals [9]. These methods range from no changes at all to building a completely new model, potentially including novel predictor variables. However, building a completely new model will discard all information contained in the prior model and is thus not the best choice for model updating [10]. Under the premise that the general model structure, i.e., the set of predictor variables and model terms such as interactions, is maintained, there are two overarching methods of model updating: recalibration and refitting [9]. Recalibration accounts for the differences in event rates and risk factor distribution in the new sample while maintaining original predictor weights. To this aim, baseline survival and mean risk factor levels are calculated from the new sample and fed to the original model. A recalibrated model therefore has the same discrimination performance, but different calibration performance compared to the original model. Refitting, on the other hand, additionally updates the predictor weights by recalculating all regression coefficients of the original model. A refitted model can therefore have different discrimination and calibration performance compared to the original model.

We now aim to validate the FRS30y in a large population-based cohort from Southern Germany (MONICA S1, N = 3110, age range 20 to 60 years) with adequate outcome ascertainment and sufficient follow-up (median follow-up 29.5 years). We compare the performance regarding discrimination and calibration for the original, recalibrated, and refitted versions of this risk score. 

## 2. Materials and Methods

### 2.1. Study Sample 

We used data from the population-based MONICA (monitoring trends and determinants in cardiovascular disease) Augsburg S1 study, sampled in 1984–1985 in the region of Augsburg, southern Germany [11]. The study is continued since then in the framework of the KORA platform (Cooperative Health Research in the Augsburg Region). Participants were followed up until 2015 for mortality, and stroke and myocardial infarction morbidity. We excluded individuals outside of the age range of 20–60 years (N = 538), individuals with prevalent MI or stroke (N = 36), coronary insufficiency (N = 110) or angina pectoris (N = 95) at baseline, and individuals with missing data on outcome (N = 54) or any of the covariates of interest (N = 68, mostly due to missing data on total cholesterol or HDL cholesterol). The final analytical sample comprised N = 3110 participants.

The study complies with the Declaration of Helsinki, including written informed consent of all participants and the follow-up was approved by the local ethics committee (Bavarian Chamber of Physicians EK No. 08064). Supplementary Table S1 shows the STROBE checklist for the present analysis.

### 2.2. Health Assessment

All participants underwent a standardized examination and interview, as detailed elsewhere [11]. Briefly, systolic blood pressure was measured according to the WHO MONICA manual. Total and HDL-cholesterol were determined from non-fasting blood samples by enzymatic methods (CHOD-PAP, Boehringer Mannheim, Ingelheim am Rhein, Germany) according to the MONICA manual. Weight and height were measured by standardized scales and BMI was calculated as weight in kg divided by height in m^2^. Obesity was defined as a BMI ≥ 30 kg/m^2^. Smoking and physician-diagnosed diabetes were assessed by self-report.

### 2.3. Outcome Assessment

CVD mortality was defined according to ICD-9, codes 390–459 and 798 by official death certificates. Stroke and MI incidence was assessed by self-report and validated by the participant’s physician and hospital records. Death due to competing causes was ascertained by official death certificates. Maximum follow-up was 32 years.

For analysis, only first events were considered.

### 2.4. Statistical Methods

Risks scores were computed based on the iterative calculations from Pencina et al. [8] in the lipids-based version, as published via the Excel spreadsheet (https://framinghamheartstudy.org/files/2020/08/Final_RISK_SCORE_lipids_open.xls, accessed on 26 February 2020). Calculations were implemented in R v 3.6.3 (R Core Team, Vienna, Austria) and vectorized to enable simultaneous calculation for the whole sample of participants. The 30-year CVD risk was calculated by (i) the original version as published: All relevant parameters (model coefficients, mean risk factor values and baseline survivals for CVD and competing death) from the original Framingham sample were used, (ii) a recalibrated version: mean risk factor values and baseline survivals were calculated from the MONICA S1 study, but model coefficients were kept as in the original publication, (iii) a refitted version, where all parameters were calculated from the MONICA S1 study. The calculated hazard ratios in comparison to those of the original Framingham cohort can be found in Supplementary Table S2. 

Survival (time free of fatal or non-fatal CVD event) across groups of FRS30y was visually assessed by Kaplan-Meier Curves and quantitatively evaluated by log-rank test. Discrimination performance of FRS30y was assessed by ROC and c-statistic (Area under the Curve, AUC). Calibration-in the-large was assessed by discordance, and moderate calibration was assessed by smooth calibration plots based on LOESS smoothing, and corresponding estimated calibration index (ECI). ECI ranges from 0 to 100 and is a measure of deviation from calibration, with smaller values indicating better and larger values indicating worse calibration [12].

As sensitivity analyses, we assessed performance considering only early events, defined as within the first 10 years of follow-up, and only fatal events. We furthermore separately analyzed performance in young individuals, i.e., aged ≤median age of 40 years at time of enrolment, and in individuals with obesity (BMI ≥ 30 kg/m^2^).

## 3. Results

### 3.1. Study Sample

**Figure 1:**
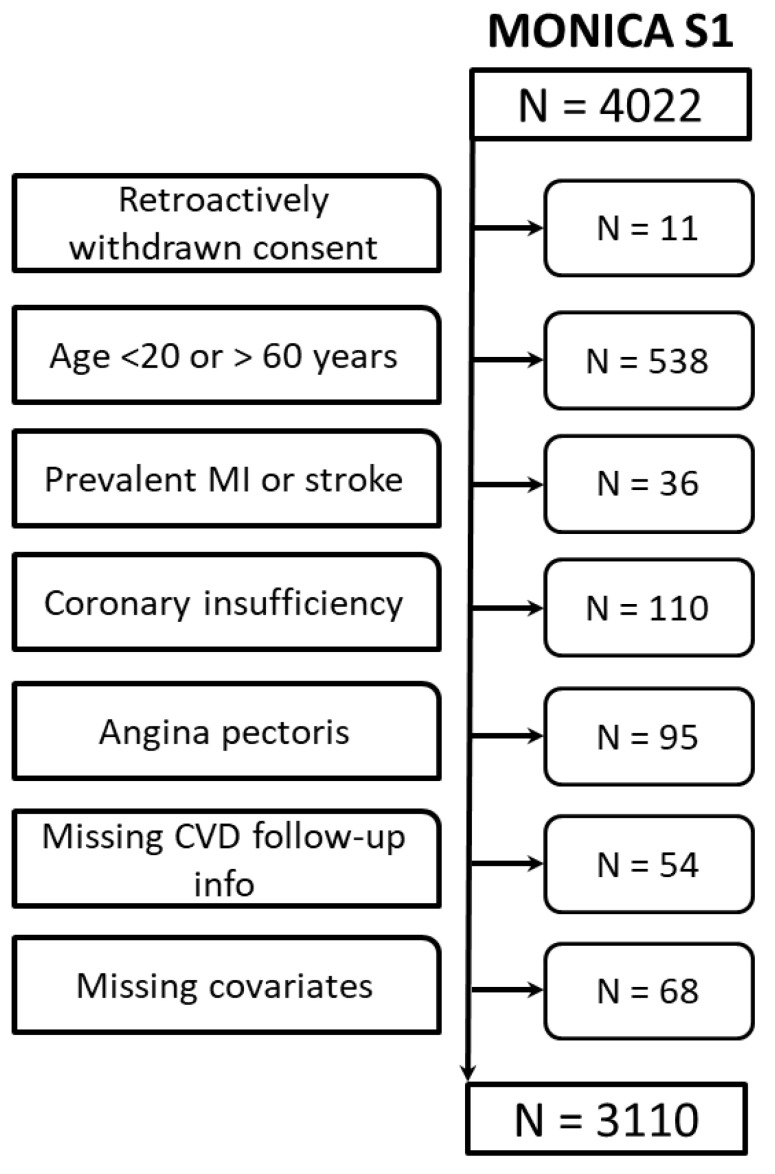
Flowchart of participants.

The study sample comprised 3110 participants, thereof 1516 (48.7%) women, as presented in Figure 1. Mean age of the participants was 42.1 years at the time of recruitment. In comparison to the original Framingham cohort our sample was on average 5 years older, had higher systolic blood pressure and total cholesterol, but also higher HDL cholesterol and a substantially lower prevalence of smoking (Supplementary Table S3). In total, 620 first CVD events (19.9%, 314 fatal) occurred during a median follow-up time of 29.5 years with pronounced differences between women and men (compare Table 1). Early events, defined as events within the first 10 years of follow-up, constituted 10.3% of all events in women, and 16.0% of all events in men. In individuals with obesity, the event rate was 33.8% in women and 38.5% in men. In individuals younger than 42 years of age, event rates were 5.5% in women and 13.9% in men. In total, 302 (9.7%) competing events, i.e., deaths due to non-CVD causes occurred.

### 3.2. Performance of FRS30y

**Figure 2:**
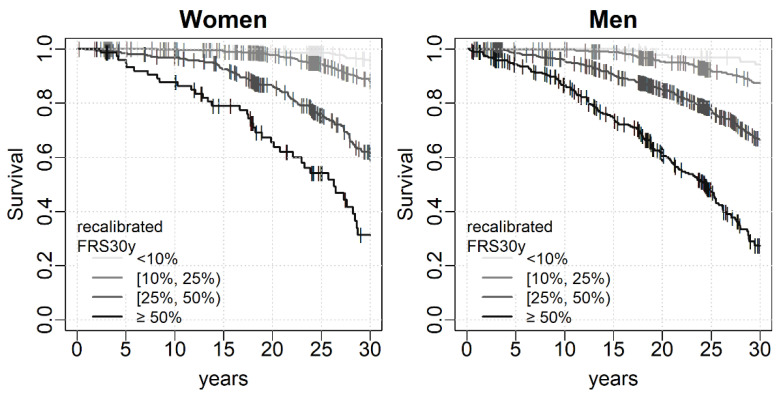
Survival (time free of fatal or non-fatal CVD event) in different risk strata. Shown are Kaplan-Meier curves for risk groups defined by different thresholds (10%, 25%, 50%) of the recalibrated FRS30y. Censored events (competing events or drop-out) are marked by vertical lines.

**Figure 3:**
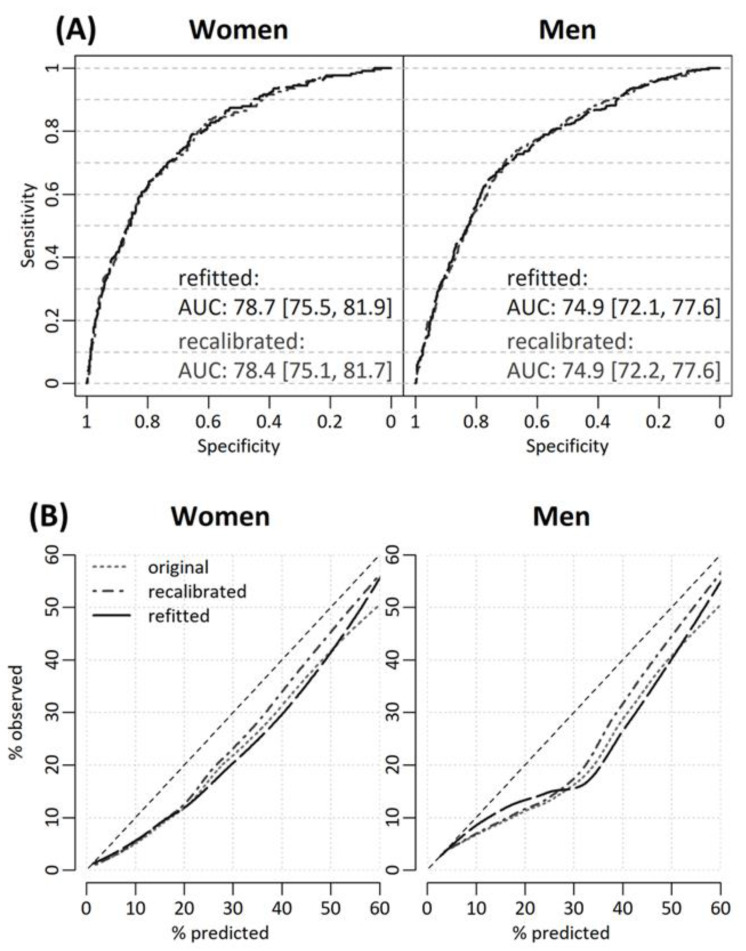
Discrimination and calibration performance of FRS30y for women and men. (**A**) upper row: ROC curves with Area Under the Curve (AUC) as measure of discrimination. As discrimination performance is not affected by recalibration, AUC values for original and recalibrated version are identical and thus the original version is not plotted. (**B**) lower row: smooth calibration based on LOESS.

Survival, defined as time free of fatal or non-fatal CVD event, was significantly different (*p* < 0.001 for both women and men, respectively) across groups of recalibrated FRS30y as shown in Kaplan-Meier curves in Figure 2. Risk strata of the original FRS30y and of the refitted FRS30y showed similar results (Supplementary Figures S1 and S2, Supplementary Table S4). Discrimination performance was better for women, however performance for both sexes was adequate (Figure 3, all AUCs > 70). The original, recalibrated, and refitted versions discriminated equally well. Calibration curves show substantial overestimation of true CVD risk for all versions and both sexes. Discordance was 45.4%, 37.6%, 44.0% in women for the original, recalibrated and refitted version respectively, while the respective values for men were 37.3%, 28.6%, and 34.5%. Generally, the recalibrated version fitted true CVD risk best for both women and men, as indicated by lowest ECI values (Table 2). 

Table 2 shows results of the main and sensitivity analyses regarding discrimination and calibration performance. Discrimination ability for early events was much higher compared to the main analyses (all AUCs > 80) and also discrimination of fatal events was better. However, calibration in both analyses was worse. Subgroup-specific analyses in individuals with obesity showed worse AUCs in women (67.6 and 68.7), but better AUCs in men (76.8 and 80.3) and generally good calibration (compare Table 2). AUCs in individuals ≤40 y were below 70 for both sexes and all FRS3 versions. 

## 4. Discussion

Using a large and well-characterized German cohort study with 30 years of CVD follow-up, we derived a recalibrated and refitted version of the FRS30y. We showed that the score has adequate discriminative ability for developing CVD over a 30-year horizon and could sensibly classify different survival strata. However, calibration could be improved, and true risk is over-estimated. To our knowledge, this is the first study to validate the FRS30y on an independent population.

As expected, both discriminative performance and calibration were worse compared to the original cohort. Framingham risk scores are known to overestimate risks in European populations [13], which might partially be due to higher disease incidence in the original cohorts and the general setup of the statistical model. We also note that risk factor distribution is substantially different between the original Framingham sample and our study sample, which will affect calibration measures [14,15]. We found that fatal and early events in particular were identified well; however, this came at the expense of major over-estimation of risk.

In our sample, the FRS30y had adequate performance after recalibration, i.e., implementing the samples risk factor values and survival rates but maintaining original model coefficients and structure. Completely refitting the model did not substantially improve discriminative performance, whereas calibration was even slightly worse for the refitted version, probably due to over- or underfitting of extreme observations. This supports the idea that the general score offers satisfactory risk prediction when adapted to the sample at hand.

The underlying model of the FRS30y is much more complex compared to risk estimates covering shorter timeframes. During 30 years of follow-up, many participants will experience non-CVD death which has to be accounted for in a competing risk component of the model. Pencina et al. showed that the competing risk component is necessary for appropriate risk prediction and that simply extrapolating from predicted 10 year risk is not permissible [8]. In the same line, a recent study on individuals aged ≥ 65 y showed that competing-risk models were superior in CVD risk prediction compared to models not accounting for competing risks [16]. Nevertheless, a recent risk score predicting 20-year CVD risk on European cohorts showed good discrimination and calibration based on a simple Cox model [17]. In the UK, the established QRISK score was expanded to yield estimates of lifetime CVD risk, providing good discrimination and calibration on UK populations [18]. This score however incorporates a variety of predictor variables that are not readily available in other studies, such as family history of disease and socioeconomic deprivation, which renders its validation in other cohorts more challenging.

We found better discriminative performance, worse calibration-in-the large (discordance) and better moderate calibration (ECI) for women. In other cohorts from our study region, we saw similar results for 10-year CVD prediction [6]. It has been shown that risk factors impact CVD risk differently according to biological sex, e.g., smoking and diabetes [19,20]. Moreover, further pathways such as inflammation [21] and systemic hormone changes [22] during the life span contribute to CVD risk in a sex-specific way. Effects of menopause on CVD risk are well known [23], but currently not captured by the FRS30y.Furthermore, we cannot exclude the possibility that CVD events were not sufficiently diagnosed in women [24] and thus the true CVD rate under-ascertained. In women with obesity, discrimination was worse compared to men with obesity, although increased BMI has been reported to confer approximately the same risk increase in both sexes [25]. More research is needed to refine sex-specific risk equations and ensure adequate calibration of risk scores for women.

In the same line, more subgroup-stratified analyses are necessary to evaluate the performance of the FRS30 in specific population groups. Thus, groups with inadequate risk prediction can be identified, and strategies can be derived in order to improve risk prediction models for these subgroups in particular. In the current study, we have presented analyses for subgroups of younger individuals or individuals with obesity, but further stratifications will be informative.

A major inherent challenge of the FRS30y is that it predicts long-term CVD risk from a static baseline set of risk factors. However, the baseline risk profile will inevitably change during the prediction horizon. The impact of risk factor changes and the effect of risk factor modifications, e.g., smoking cessation or medication treatment can thus not be captured by a one-time calculation of the FRS30y. Correspondingly, the investigators of the original FRS30y found changes in hazard ratio estimates when risk factors were model to be time-dependent [8]. Substantial differences were observed for changes in for smoking behavior [8], which can be explained by the fact that smoking cessation leads to a relatively abrupt decrease in short-term CVD risk [26]. There already are efforts to incorporate changes in treatment regimens into lifetime CVD risk prediction [27]. However, the use of longitudinal risk factor trajectories, which would incorporate changing risk factor levels and hence have potential for improved prediction, still has to be implemented in a clinically applicable risk score. 

Moreover, the FRS30y is based on a restricted set of traditional risk factors, which are readily available in clinical practice. Cardiovascular risk is also impacted by genetics [28], environmental factors such as air pollution, noise, or heat [29], and interactions thereof [30], as well as by psychosocial and socioeconomic factors, such as mental well-being, chronic stress or economic deprivation [31]. However, these factors are not explicitly mapped in the FRS30y but only captured by mediation through the included set of traditional risk factors. 

The importance of risk factor levels in early adulthood for subsequent lifetime CVD mortality has already been recognized [32]. Given the modifiable nature of many CVD risk factors, early prevention is feasible, especially for behavioral changes such as dietary amendments, physical activity and smoking cessation. Early interventions are desirable and can result in substantially improved health trajectories [33]. A recent study on young hypertensive patients showed substantial CVD risk improvement for those adhering to antihypertensive medication compared to those who did not [34]. In clinical practice, we see the main utility of the FRS30y in risk communication: Communicating a long-term risk quantification such as provided by the FRS30y could lead to a better understanding of the potential consequences of persistent detrimental behaviors and subsequent unfavorable risk factor profiles, especially for younger patients. Furthermore, communicating a combination of both 10-year and 30-year CVD risk prediction could be useful for a more complete picture of CVD risk. By repeated calculations of the FRS30y at different time points in life, trajectories of long-term CVD risk, and effects of treatment initiations and lifestyle modifications can be effectively monitored.

To enable individual access to this long-term risk prediction, a digital application, incorporating the recalibrated FRS30y, will be designed in the framework of the *DigiMed Bayern* *Consortium* with focus on personalization and digitalization in cardiovascular medicine. As a daily companion, giving individualized lifestyle recommendations, the app might help to improve CVD prevention beyond conventional medical contact.

Our study has limitations. Most importantly, the baseline data are inevitably quite old, as the underlying cohort was enrolled in the mid-eighties. Thus, risk factor distributions, treatment guidelines, and outcome ascertainment are different from current cohorts. In recent years, guidelines for the treatment of dyslipidemia [35] and hypertension [36] have notably changed, which will be reflected in risk factor levels and prevalence of medication intake in contemporary cohorts. This will likely have especially affected the calibration results, as calibration drift over time is commonly seen in risk prediction models [14]. Moreover, our sample, just like the Framingham cohort, represents a restricted population of white ethnicity and thus its generalizability is limited.

In conclusion, the performance of the FRS30y is adequate for long-term CVD risk prediction in a German population-based sample and could serve as an important tool in risk communication, especially for younger audiences.

## Figures and Tables

**Table 1:** Baseline characteristics of study sample and estimated risk by original, recalibrated and refitted version of FRS30y and true event rates. Data are given as mean ± standard deviation for continuous covariates, unless otherwise indicated. Data are given as counts (percentage) for categorical covariates. *p*-values from *t*-Test or χ^2^-Test, where appropriate.

	Women	Men	*p*-Value
	N = 1516	N = 1594	
**Risk Factor Distribution**			
Age, years	42.0 ± 9.6	42.2 ± 9.9	0.44
BMI, kg/m^2^	25.4 ± 4.5	26.7 ± 3.5	<0.001
Obesity (BMI ≥ 30)	222 (14.6%)	257 (16.1%)	0.275
Systolic Blood Pressure, mmHg	123.9 ± 17.3	132.4 ± 15.8	<0.001
Antihypertensive Treatment	92 (6.1%)	66 (4.1%)	0.018
Total Cholesterol, mg/dL	223.9 ± 44.6	234.1 ± 46.4	<0.001
HDL Cholesterol, mg/dL	64.3 ± 17.4	51.0 ± 15.4	<0.001
Lipid-lowering Treatment	5 (0.3%)	19 (1.2%)	0.011
Diabetes, self-reported	13 (0.9%)	26 (1.6%)	0.076
Smoking	373 (24.6%)	630 (39.5%)	<0.001
**True event rates**			
CVD event	214 (14.1%)	406 (25.5%)	<0.001
Early CVD event (within 10y of follow-up)	22 (1.5%)	65 (4.1%)	<0.001
Fatal CVD event	115 (7.6%)	199 (12.5%)	<0.001
Competing event	117 (7.7%)	185 (11.6%)	<0.001
Follow-up time, years (median [1st quartile, 3rd quartile])	30.9 [21.8, 31.2]	26.7 [17.7, 31.2]	<0.001
**Estimated CVD event risk**			
FRS30y, in %			
original	20.5 ± 15.9	35.0 ± 19.5	<0.001
recalibrated	19.4 ± 15.0	32.8 ± 17.9	<0.001
refitted	20.3 ± 17.0	34.3 ± 20.4	<0.001

**Table 2:** Discrimination (values as AUC) and calibration performance (values as ECI) of different FRS30y versions for main and sensitivity analyses. As recalibration does not affect discrimination performance, the original and recalibrated versions are subsumed presentation of discrimination performance.

	Discrimination (AUC)				Calibration (ECI)					
	Women		Men		Women			Men		
	Original/ recalibrated	refitted	Original/ recalibrated	refitted	original	recalibrated	refitted	original	recalibrated	refitted
Main analysis	78.4	78.7	74.9	74.9	0.511	0.343	0.524	1.086	0.659	1.064
**Sensitivity analysis on specific events**										
Early events (within 10 y of FU)	84	84.2	82	84.5	5.548	4.910	5.710	11.757	10.046	11.553
Fatal events	79.2	80	78.1	79.9	2.348	1.955	2.369	5.883	4.764	5.734
**Sensitivity analysis on specific subgroups**										
BMI ≥ 30 kg/m^2^	67.6	68.7	76.8	80.3	0.420	0.292	0.304	0.637	0.253	0.596
Age ≤ 40 y	69.6	69.6	69.8	69.8	0.409	0.342	0.226	1.247	0.984	0.750

## Data Availability

The informed consent given by KORA study participants does not cover data posting in public databases. However, data are available upon request by means of a project agreement. Requests should be sent to kora.passt@helmholtz-muenchen.de and are subject to approval by the KORA Board.

## Supplementary Materials

The following supporting information can be downloaded at: https://www.mdpi.com/article/10.3390/diagnostics12040965/s1, Supplementary Table S1: STROBE Statement—Checklist of items that should be included in reports of cohort studies. Supplementary Table S2: Hazard Ratios for outcome CVD of individual risk factors in the Framingham and MONICA S1 study. Supplementary Table S3: Risk factor distribution in the Framingham and MONICA S1 study. Supplementary Figure S1: Survival in different risk strata, as defined by the original FRS30y. Supplementary Figure S2: Survival in different risk strata, as defined by the refitted FRS30y. Supplementary Table S4: Differences in survival according to risk strata. Supplementary Text S1: Members of The DigiMed Bayern Consortium.
