# Supplementary material for: Validation of the 30-Year Framingham Risk Score in a German Population-Based Cohort
Source: Diagnostics (Basel). 2022 Apr 12;12(4):965. doi: 10.3390/diagnostics12040965 (PMC9025787; doi:10.3390/diagnostics12040965)
Supplement: Supplementary file 1 [file diagnostics-12-00965-s001.zip › diagnostics-1654731-supplementary.pdf]

Supplementary Material to *Rospleszcz et al "Validation of the 30-year Framingham Risk Score in a German population-based cohort"*

Supplementary Table S1: STROBE Statement—Checklist of items that should be included in reports of ***cohort studies***

	Item No	Recommendation	Page No
**Title and abstract**	1	(*a*) Indicate the study's design with a commonly used term in the title or the abstract	1
		(*b*) Provide in the abstract an informative and balanced summary of what was done and what was found	3
**Introduction**			
Background/rationale	2	Explain the scientific background and rationale for the investigation being reported	5
Objectives	3	State specific objectives, including any prespecified hypotheses	6
**Methods**			
Study design	4	Present key elements of study design early in the paper	7
Setting	5	Describe the setting, locations, and relevant dates, including periods of recruitment, exposure, follow-up, and data collection	7-8
Participants	6	(*a*) Give the eligibility criteria, and the sources and methods of selection of participants. Describe methods of follow-up	8
		(*b*) For matched studies, give matching criteria and number of exposed and unexposed	n/a
Variables	7	Clearly define all outcomes, exposures, predictors, potential confounders, and effect modifiers. Give diagnostic criteria, if applicable	7
Data sources/ measurement	8*	For each variable of interest, give sources of data and details of methods of assessment (measurement). Describe comparability of assessment methods if there is more than one group	7
Bias	9	Describe any efforts to address potential sources of bias	9
Study size	10	Explain how the study size was arrived at	9, 19
Quantitative variables	11	Explain how quantitative variables were handled in the analyses. If applicable, describe which groupings were chosen and why	8
Statistical methods	12	(*a*) Describe all statistical methods, including those used to control for confounding	8
		(*b*) Describe any methods used to examine subgroups and interactions	9
		(*c*) Explain how missing data were addressed	9
		(*d*) If applicable, explain how loss to follow-up was addressed	n/a
		(*e*) Describe any sensitivity analyses	9
**Results**			
Participants	13*	(a) Report numbers of individuals at each stage of study—eg numbers potentially eligible, examined for eligibility, confirmed eligible, included in the study, completing follow-up, and analysed	19
		(b) Give reasons for non-participation at each stage	n/a

		(c) Consider use of a flow diagram	19
Descriptive data	14*	(a) Give characteristics of study participants (eg demographic, clinical, social) and information on exposures and potential confounders	17
		(b) Indicate number of participants with missing data for each variable of interest	n/a
		(c) Summarise follow-up time (eg, average and total amount)	8, 9
Outcome data	15*	Report numbers of outcome events or summary measures over time	9, 17

Main results	16	(*a*) Give unadjusted estimates and, if applicable, confounder-adjusted estimates and their precision (eg, 95% confidence interval). Make clear which confounders were adjusted for and why they were included	9, 17
		(*b*) Report category boundaries when continuous variables were categorized	n/a
		(*c*) If relevant, consider translating estimates of relative risk into absolute risk for a meaningful time period	n/a
Other analyses	17	Report other analyses done—eg analyses of subgroups and interactions, and sensitivity analyses	18
**Discussion**			
Key results	18	Summarise key results with reference to study objectives	11
Limitations	19	Discuss limitations of the study, taking into account sources of potential bias or imprecision. Discuss both direction and magnitude of any potential bias	13
Interpretation	20	Give a cautious overall interpretation of results considering objectives, limitations, multiplicity of analyses, results from similar studies, and other relevant evidence	13
Generalisability	21	Discuss the generalisability (external validity) of the study results	12-13
**Other information**			
Funding	22	Give the source of funding and the role of the funders for the present study and, if applicable, for the original study on which the present article is based	2

*Give information separately for exposed and unexposed groups.

Note: An Explanation and Elaboration article discusses each checklist item and gives methodological background and published examples of transparent reporting. The STROBE checklist is best used in conjunction with this article (freely available on the Web sites of PLoS Medicine at http://www.plosmedicine.org/, Annals of Internal Medicine at http://www.annals.org/, and Epidemiology at http://www.epidem.com/). Information on the STROBE Initiative is available at http://www.strobe-statement.org.

Supplementary Table S2: Hazard Ratios for outcome CVD of individual risk factors in the Framingham and MONICA S1 study.

**Risk factor**	**Framingham**	**MONICA**
Male sex	1.73 (1.45, 2.07)	1.53 (1.27, 1.83)
Age	2.09 (1.88, 2.31)	2.32 (2.07, 2.60)
SBP	1.29 (1.19, 1.39)	1.33 (1.22, 1.44)
Antihypertensive treatment	1.48 (1.10, 2.00)	1.53 (1.17, 2.00)
Smoking	2.01 (1.72, 2.35)	1.89 (1.60, 2.24)
Diabetes mellitus	2.49 (1.82, 3.41)	2.98 (1.96, 4.51)
Total cholesterol	1.33 (1.23, 1.44)	1.19 (1.09, 1.30)
HDL cholesterol	0.78 (0.72, 0.84)	0.83 (0.76, 0.89)

Values denote hazard ratios with corresponding 95% confidence interval. Hazard ratios for continuous risk factors are given per 1 SD increase in the natural logarithm. Values for the Framingham cohort adapted from Table 2 in [1].

Supplementary Table S3: Risk factor distribution in the Framingham and MONICA S1 study

	Framingham		MONICA	
	**Women** N = 2333	**Men** N = 2173	**Women** N = 1516	**Men** N = 1594
**Risk factor distribution**				
Age, years	36.3 ± 9.3	37.3 ± 9.2	42.0 ± 9.6	42.2 ± 9.9
SBP, mmHg	118 ± 16	126 ± 15	123.9 ± 17.3	132.4 ± 15.8
Antihypertensive treatment, %	2.7	3.1	6.1	4.1
Total cholesterol, mg/dL	192 ± 38	202 ± 39	223.9 ± 44.6	234.1 ± 46.4
HDL cholesterol, mg/dL	57 ± 15	44 ± 12	64.3 ± 17.4	51.0 ± 15.4
BMI, kg/m^2	23.9 ± 4.5	26.5 ± 3.6	25.4 ± 4.5	26.7 ± 3.5
Smoking, %	45	46.2	24.6	39.5
Diabetes mellitus, %	0.9	2.6	0.9	1.6
Incident events, n	219	452	214	406

Values are mean±SD for continuous variables and numbers or percentages for categorical variables. Values for the Framingham cohort adapted from Table 1 in [1].

Supplementary Figure S1: Survival (time free of fatal or non-fatal CVD event) in different risk strata, as defined by the original FRS30y. Shown are Kaplan-Meier curves for risk groups defined by different thresholds (10%, 25%, 50%) of the original FRS30y. Censored events (competing events or drop-out) are marked by vertical lines.

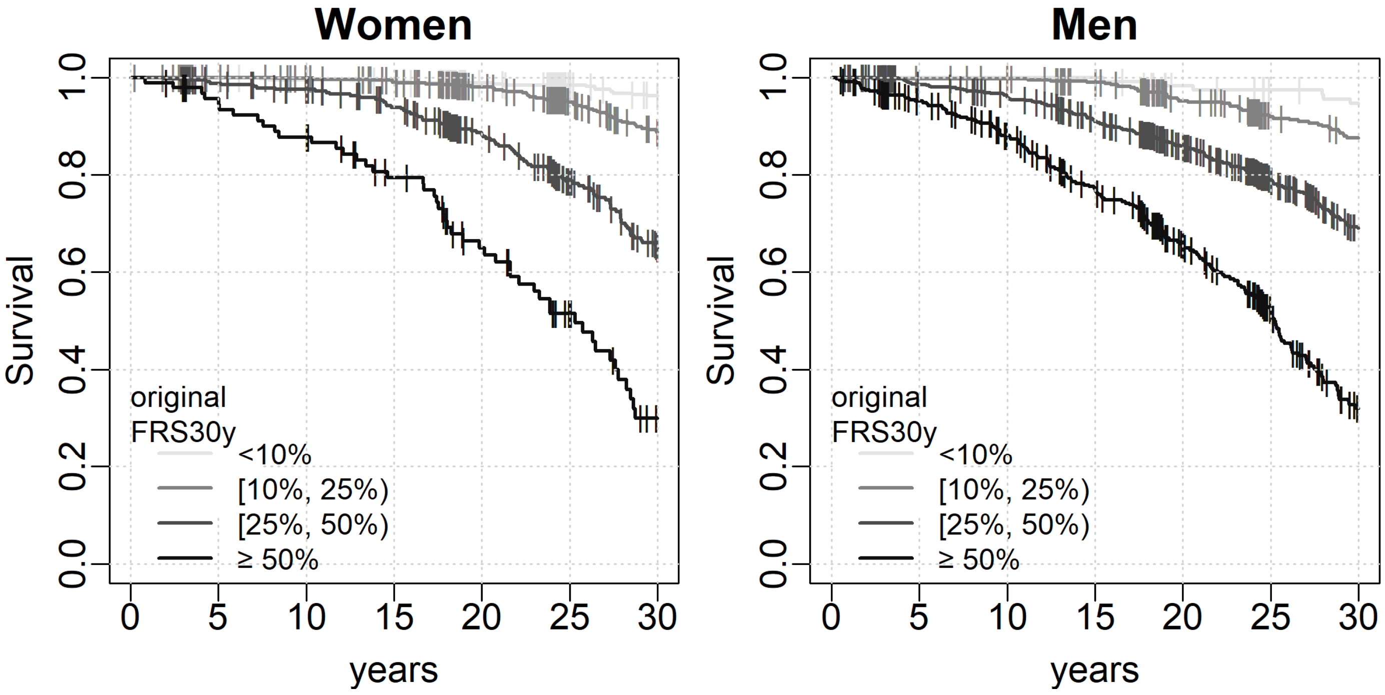

Supplementary Figure S2: Survival (time free of fatal or non-fatal CVD event) in different risk strata, as defined by the refitted FRS30y. Shown are Kaplan-Meier curves for risk groups defined by different thresholds (10%, 25%, 50%) of the refitted FRS30y. Censored events (competing events or drop-out) are marked by vertical lines.

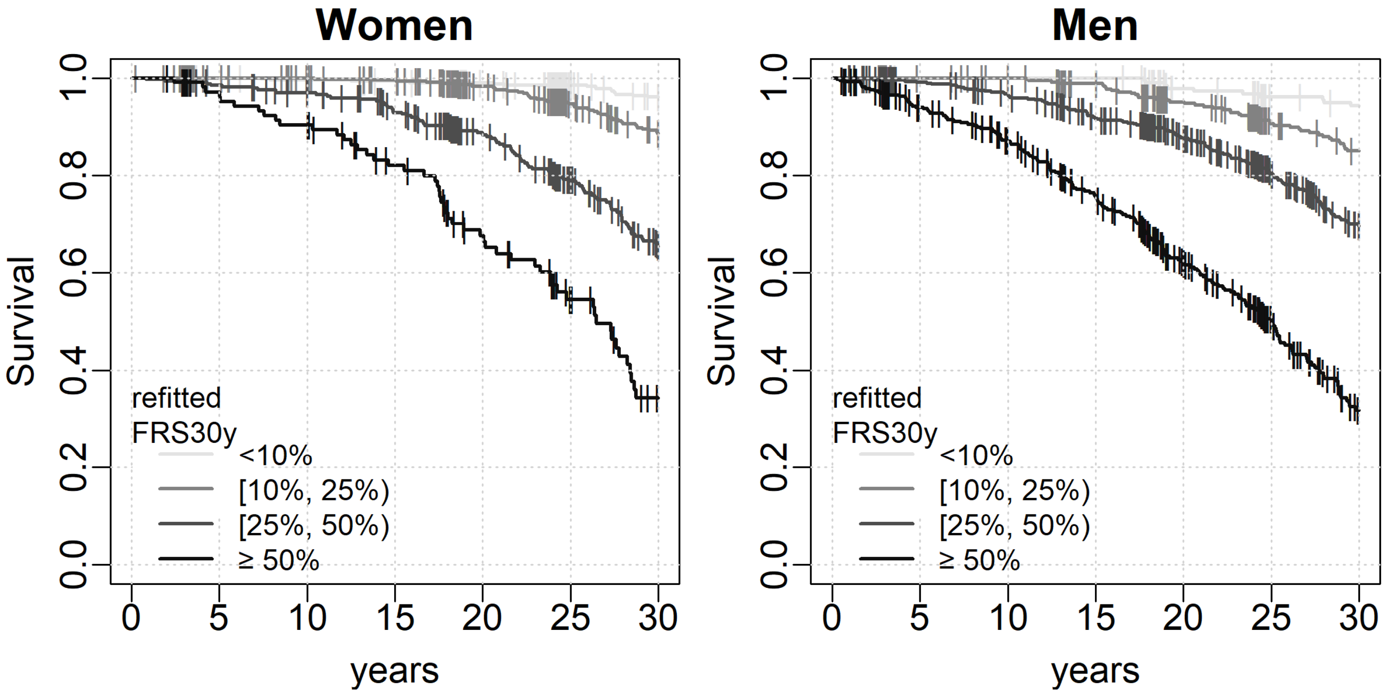

Supplementary Table S4: Differences in survival according to risk strata.

	Women	Men
recalibrated	9.29E-77	2.13E-71
original	2.61E-84	5.64E-67
refitted	2.11E-78	3.09E-76

Shown are p-values from a log-rank test according to the risk strata <10%, [10%, 25%), [25%, 50%), ≥50%, if defined by the recalibrated, original or refitted version of the FRS30y.

Supplementary Text S1: Members of The DigiMedBayernConsortium

Jonathan Adam, Institut für Medizinische Informationsverarbeitung Biometrie und Epidemiologie, Ludwig-Maximilians-Universität München, Munich, Germany; Institut für Epidemiologie, Helmholtz Zentrum München, Munich, Germany; Reiner Anselm, Institut Technik – Theologie – Naturwissenschaften, Ludwig-Maximilians-Universität München, Munich, Germany; Sara Ates, Deutsches Herzzentrum München, Klinik für Herz- und Kreislauferkrankungen, Technische Universität München, Munich, Germany; Sabine Bauer, Deutsches Herzzentrum München, Klinik für Herz- und Kreislauferkrankungen, Technische Universität München, Munich, Germany; Nicole Beck, Deutsches Herzzentrum München, Klinik für Herz- und Gefäßchirurgie, Technische Universität München, Munich, Germany; Jürgen Beckmann, Fakultät für Sport- und Gesundheitswissenschaften, Technische Universität München, Munich, Germany; Riccardo Berutti, Institut für Neurogenomik, Helmholtz Zentrum München, Munich, Germany; Stefan Brandmaier, Institut für Epidemiologie, Helmholtz Zentrum München, Munich, Germany; Theresa Brunet, Institut für Humangenetik, Technische Universität München, Munich, Germany; Salvatore Cassese, Deutsches Herzzentrum München, Klinik für Herz- und Kreislauferkrankungen, Technische Universität München, Munich, Germany; Manuela Decker, Deutsches Herzzentrum München, Klinik für Herz- und Kreislauferkrankungen, Technische Universität München, Munich, Germany; Martin Dichgans, Institut für Schlaganfall- und Demenzforschung, Ludwig-Maximilians-Universität München, Munich, Germany; Philine Diesselhorst, Institut Technik – Theologie – Naturwissenschaften, Ludwig-Maximilians-Universität; Horst Domdey, BioM Biotech Cluster Development GmbH, Martinsried, Germany; Martina Dreßen, Deutsches Herzzentrum München, Klinik für Herz- und Gefäßchirurgie, Technische Universität München, Munich, Germany; Arne Dressler, Institut Technik – Theologie – Naturwissenschaften, Ludwig-Maximilians-Universität München, Munich, Germany; Florent Dufour, Leibniz-Rechenzentrum, Munich, Germany; Sven Duscha, Deutsches Herzzentrum München, Klinik für Herz- und Kreislauferkrankungen, Technische Universität München, Munich, Germany; Hans-Henning Eckstein, Klinik und Poliklinik für Vaskuläre und Endovaskuläre Chirurgie, Klinikums rechts der Isar, , Technische Universität München, Munich, Germany; Aiman Farzeen, Institut für Humangenetik, Technische Universität München, Munich, Germany; Institut für Epidemiologie, Helmholtz Zentrum München, Munich, Germany; Therese Feiler, Institut Technik – Theologie – Naturwissenschaften, Ludwig-Maximilians-Universität München, Munich, Germany; Ines Gall, Deutsches Herzzentrum München, Institut für Laboratoriumsmedizin, Technische Universität München, Munich, Germany; Ulrich M. Gassner, Juristische Fakultät, Universität Augsburg, Augsburg, Germany; Christian Gieger, Institut für Epidemiologie, Helmholtz Zentrum München, Munich, Germany; Monica Gotor-Blazquez, Deutsches Herzzentrum München, Klinik für Herz- und Kreislauferkrankungen, Technische Universität München, Munich, Germany; Ulrich Güldener, Deutsches Herzzentrum München, Klinik für Herz- und Kreislauferkrankungen, Technische Universität München, Munich, Germany; Nicolay Hammer, Max-Planck-Institut für Biochemie, Munich, Germany; Johann Hawe, Deutsches Herzzentrum München, Klinik für Herz- und Kreislauferkrankungen, Technische Universität München, Munich, Germany; Thomas Hendel, Institut für Epidemiologie, Helmholtz Zentrum München, Munich, Germany; Stefan Holdenrieder; Deutsches Herzzentrum München, Institut für Laboratoriumsmedizin, Technische Universität München, Munich, Germany; Stephan Jonas, Institut für Informatik, Technische Universität München, Munich, Germany; Adnan Kastrati, Deutsches Herzzentrum München, Klinik für Herz- und Kreislauferkrankungen, Technische Universität München, Munich, Germany; Wolfgang Kempf, Klinikums rechts der Isar, Technische Universität München, Munich, Germany; Thorsten Keßler, Deutsches Herzzentrum München, Klinik für Herz- und Kreislauferkrankungen, Technische Universität München, Munich, Germany; Wolfgang Koenig, Deutsches Herzzentrum München, Klinik für Herz- und Kreislauferkrankungen, Technische Universität München, Munich, Germany; Florian Kohlmayer, Bitcare, Munich, Germany; Markus Krane, Deutsches Herzzentrum München, Klinik für Herz- und Gefäßchirurgie, Technische Universität München, Munich, Germany; Dieter Kranzlmüller, Institut für Informatik, Ludwig-Maximilians-Universität München, Munich, Germany; Harald Lahm, Deutsches Herzzentrum München, Klinik für Herz- und Gefäßchirurgie, Technische Universität München, Munich, Germany; Rüdiger Lange, Deutsches Herzzentrum München, Klinik für Herz- und Gefäßchirurgie, Technische Universität München, Munich, Germany; Andreas Lehmann, Bitcare GmbH, Munich, Germany; Ling Li, Deutsches Herzzentrum München, Klinik für Herz- und Kreislauferkrankungen, Technische Universität München, Munich, Germany; Birgit Linkohr, Institut für Epidemiologie, Helmholtz Zentrum München, Munich, Germany; Lars Maegdefessel, Klinik und Poliklinik für Vaskuläre und Endovaskuläre

Chirurgie, Klinikums rechts der Isar, Technische Universität München, Munich, Germany; Matthias Mann, Max-Planck-Institut für Biochemie, Munich, Germany; Rainer Malik, Institut für Schlaganfall- und Demenzforschung, Ludwig-Maximilians-Universität München, Munich, Germany; Thomas Meitinger, Institut für Humangenetik, Technische Universität München, Munich, Germany; Irina Neb, Deutsches Herzzentrum München, Klinik für Herz- und Gefäßchirurgie, Technische Universität München, Munich, Germany; Tina O'Hehir, Deutsches Herzzentrum München, Klinik für Herz- und Kreislauferkrankungen, Technische Universität München, Munich, Germany; Shichao Pang, Deutsches Herzzentrum München, Klinik für Herz- und Kreislauferkrankungen, Technische Universität München, Munich, Germany; Benedikt Perl, Fakultät für Sport- und Gesundheitswissenschaften, Technische Universität München, Munich, Germany; Annette Peters, Institut für Medizinische Informationsverarbeitung Biometrie und Epidemiologie Ludwig-Maximilians-Universität München, Munich, Germany; Institut für Epidemiologie, Helmholtz Zentrum München, Munich, Germany; Fatemeh Peymani, Institut für Humangenetik, Technische Universität München, Munich, Germany; Roland Pichler, Max-Planck-Institut für Biochemie, Munich, Germany; Heiko Pfister, Deutsches Herzzentrum München, Institut für Laboratoriumsmedizin, Technische Universität München, Munich, Germany; Paola Pisano, Max-Planck-Institut für Biochemie, Munich, Germany; Holger Prokisch, Institut für Humangenetik, Technische Universität München, Munich, Germany; Lara Marie Reimer, Institut für Informatik, Technische Universität München, Munich, Germany; Michaela Sander, Deutsches Herzzentrum München, Institut für Laboratoriumsmedizin, Technische Universität München, Munich, Germany; Veronika Sanin, Deutsches Herzzentrum München, Klinik für Herz- und Kreislauferkrankungen, Technische Universität München, Munich, Germany; Lea Dewi Schlieben, Institut für Humangenetik, Technische Universität München, Munich, Germany; Yannick Schlote, Institut Technik – Theologie – Naturwissenschaften, Ludwig-Maximilians-Universität München, Munich, Germany; Sofie Schmid, Klinikums rechts der Isar, Technische Universität München, Munich, Germany; Raphael Schmieder, Deutsches Herzzentrum München, Klinik für Herz- und Kreislauferkrankungen, Technische Universität München, Munich, Germany; Heribert Schunkert, Deutsches Herzzentrum München, Klinik für Herz- und Kreislauferkrankungen, Technische Universität München, Munich, Germany; Megi Sharikadze, Leibniz-Rechenzentrum, Munich, Germany; Ankit Sinha, Max-Planck-Institut für Biochemie, Munich, Germany; Fabian Starnecker, Deutsches Herzzentrum München, Klinik für Herz- und Kreislauferkrankungen, Technische Universität München, Munich, Germany; Medini Steger, Max-Planck-Institut für Biochemie, Munich, Germany; Sophia Steigerwald, Max-Planck-Institut für Biochemie, Munich, Germany; Ruoyu Sun, BioM Biotech Cluster Development GmbH, Martinsried, Germany; Moritz von Scheidt, Deutsches Herzzentrum München, Klinik für Herz- und Kreislauferkrankungen, Technische Universität München, Munich, Germany; Matias Wagner, Institut für Humangenetik, Technische Universität München, Munich, Germany; Annie Westerlund, Deutsches Herzzentrum München, Klinik für Herz- und Kreislauferkrankungen, Technische Universität München, Munich, Germany; Jens Wiehler, BioM Biotech Cluster Development GmbH, Martinsried, Germany; Michael Wierer, Max-Planck-Institut für Biochemie, Munich, Germany; Peter Zinterhof, Leibniz-Rechenzentrum, Munich, Germany

Supplementary References

1. Pencina, M.J.; D'Agostino, R.B., Sr.; Larson, M.G.; Massaro, J.M.; Vasan, R.S. Predicting the 30-year risk of cardiovascular disease: the framingham heart study. *Circulation* **2009**, *119*, 3078-3084, doi:10.1161/CIRCULATIONAHA.108.816694.
